# Effect of Pregabalin on Cardiovascular Responses to Exercise and Postexercise Pain and Fatigue in Fibromyalgia: A Randomized, Double-Blind, Crossover Pilot Study

**DOI:** 10.1155/2015/136409

**Published:** 2015-12-29

**Authors:** Andrea T. White, Kathleen C. Light, Lucinda Bateman, Ronald W. Hughen, Timothy A. Vanhaitsma, Alan R. Light

**Affiliations:** ^1^Department of Anesthesiology, University of Utah, Salt Lake City, UT 84112, USA; ^2^Department of Exercise and Sport Science, University of Utah, Salt Lake City, UT 84112, USA; ^3^Bateman Horne Center of Excellence, Salt Lake City, UT 84102, USA; ^4^Department of Kinesiology, Westmont College, Santa Barbara, CA 93108, USA

## Abstract

Pregabalin, an approved treatment for fibromyalgia (FM), has been shown to decrease sympathetic nervous system (SNS) activity and inhibit sympathetically maintained pain, but its effects on exercise responses have not been reported.* Methods*. Using a randomized double-blind crossover design, we assessed the effect of 5 weeks of pregabalin (versus placebo) on acute cardiovascular and subjective responses to moderate exercise in 19 FM patients. Blood pressure (BP), heart rate (HR), and ratings of perceived exertion (RPE) during exercise and ratings of pain, physical fatigue, and mental fatigue before, during, and for 48 hours after exercise were compared in patients on pregabalin versus placebo and also versus 18 healthy controls.* Results*. On placebo, exercise RPE and BP were significantly higher in FM patients than controls (*p* < 0.04). Pregabalin responders (*n* = 12, defined by patient satisfaction and symptom changes) had significantly lower exercise BP, HR, and RPE on pregabalin versus placebo (*p* < 0.03) and no longer differed from controls (*p* > 0.26). Cardiovascular responses of nonresponders (*n* = 7) were not altered by pregabalin. In responders, pregabalin improved ratings of fatigue and pain (*p* < 0.04), but negative effects on pain and fatigue were seen in nonresponders.* Conclusions*. These preliminary findings suggest that pregabalin may normalize cardiovascular and subjective responses to exercise in many FM patients.

## 1. Introduction

Fibromyalgia syndrome (FM) is characterized by widespread pain in muscles, joints, and deep connective tissues for at least three months. Additionally, using the American College of Rheumatology (ACR) classification criteria, FM patients report painful hypersensitivity to pressure in at least 11 of 18 tender points during clinical examination [1]. FM patients represent a heterogeneous population of chronic pain patients, making them challenging to diagnose and to treat successfully [2]. One example of this heterogeneity is that many FM patients have comorbid pain and fatigue disorders, such as myalgic encephalomyelitis/chronic fatigue syndrome (ME/CFS, hereafter referred to as CFS), migraine headaches, irritable bowel syndrome, and temporomandibular disorder, while other FM patients do not [3–5].

Heterogeneity among FM patients is also reflected in their differing response to medications, including pregabalin, the first drug licensed by the U.S. Food and Drug Administration (FDA) specifically for treatment of FM [6]. Pregabalin is a potent inhibitory ligand for *α*2-delta subunit of calcium channels in the central nervous system (CNS) that has analgesic and anxiolytic as well as anticonvulsant activity. Clinical trials of pregabalin have shown significant reduction of pain, improved sleep quality, and improved health-related quality of life in the majority of FM patients studied [6–8]. However, a common side effect of pregabalin is an increase in lethargy and sedation, which is especially problematic for FM patients with comorbid CFS who already have profound physical and mental fatigue (mental fog). Other common side effects that deter patients from continuing pregabalin use include weight gain, swelling and tingling in extremities, and dizziness. Thus, Smith and Moore [9] conclude that pregabalin has a therapeutic benefit above placebo with tolerable side effects in only approximately 50% of FM patients (pregabalin responders) while the other 50% of FM patients are nonresponders to this treatment.

It is also notable that exercise therapy (both aerobic and strength training) has been shown to reduce pain ratings and increase function in FM patients [10–12]. However, because many FM patients (especially those with comorbid CFS) experience postexertional worsening of pain, physical fatigue, and mental fog, the dropout rate of FM patients from exercise programs is high [13, 14]. This postexertional symptom worsening is thus important not just because of its impact on the person's symptoms during the next several days following each bout of exercise but also because it serves as a strong and immediate disincentive to exercise [15] and contributes to long term physical deconditioning [14]. Thus, it is clinically important to determine whether medications for FM such as pregabalin may moderate these postexercise symptoms.

To our knowledge, no previous studies have used standardized exercise tasks with extended postexercise symptom monitoring to assess effects of pregabalin versus placebo on cardiovascular responses or symptom severity in patients with FM. The most similar investigation that we could find was a study of postexercise changes in pain ratings in CFS + FM patients, rheumatoid arthritis patients, and healthy controls on placebo versus paracetamol. That study found mixed responses in CFS + FM patients, with paracetamol associated with decreased pain immediately after exercise in some but not all of these patients [16]. In our previous studies on patients with comorbid CFS + FM [17, 18], we had determined* post hoc *that patients tested while on their normal medications that included pregabalin had lower pain and mental fatigue scores both at preexercise baseline and for at least 48 hours after acute exercise than patients on other treatments (unpublished data). In these studies, the acute exercise task consisted of 25 minutes of combined arm and leg cycling at 70% of age-predicted maximal heart rate. Using this information, we hypothesized that pregabalin treatment would decrease pain and fatigue symptoms in at least some patients both at preexercise baseline and following an acute exercise challenge. We further hypothesized that pregabalin treatment would partially normalize cardiovascular differences in response to exercise seen between FM patients and healthy controls, with the caveat that this beneficial effect might be seen only in those patients who are treatment responders and/or those patients without comorbid CFS.

## 2. Methods

### 2.1. Participants

Written informed consent approved by the University of Utah Institutional Review Board was obtained from all study volunteers before participation. Our objective was to recruit 20 patients aged 18–65 from a private fatigue clinic who met the 1990 ACR criteria for FM [1] and who were not currently using pregabalin or a related anticonvulsant drug, gabapentin, or opioids to treat their pain. Current use of serotonin- and norepinephrine-reuptake inhibitors was not exclusionary [1]. Of these 20 FM patients, we intended to recruit 10 who also met both Fukuda et al.'s 1994 case definition [19] and the Canadian criteria for myalgic encephalomyelitis/chronic fatigue syndrome (CFS + FM). Sample size was estimated based on prior data showing gene expression differences between CFS/FM patients while taking pregabalin or gabapentin versus patients on neither drug [17]. Healthy age- and gender-matched controls (*n* = 18) were also recruited for participation in the exercise protocol as a comparison group. Data were collected between June 2011 and June 2013.

### 2.2. Study Protocol

This pilot study utilized a double-blind, placebo-controlled crossover design to examine pre- and postexercise leukocyte gene expression changes induced by pregabalin (Lyrica) in patients with FM, patients with both CFS + FM, and healthy controls. Evaluation of symptom changes, subjective ratings of pain and fatigue, and responses to an acute exercise challenge were obtained in conjunction with treatment and placebo conditions. Here, we report on cardiovascular and self-reported symptom responses to exercise with respect to treatment effects, while the gene expression results will be reported in a separate paper (Light et al., in preparation). Pregabalin and identical appearing placebo were provided by Pfizer.

### 2.3. Drug Treatment and Effects

Upon study entry, patients were examined (by Lucinda Bateman) at the Fatigue Consultation Clinic (FCC), where the diagnosis of CFS + FM or FM was confirmed. Patients were randomized to receive either pregabalin or placebo for 5 weeks, including a 2-week upward titration phase up to 450 mg/day. FM and CFS + FM groups were randomized in blocks in order to achieve similar numbers in each group who received pregabalin or placebo treatment first. FCC staff were not blinded to the treatment the patients received for safety reasons. At the end of 5 weeks, patients reported for exercise testing in the Department of Anesthesia at the University of Utah, as described below. All University of Utah research staff, including the PI, were blind to the diagnosis and treatment of the patients. Following a 2-week washout period supervised by FCC staff, patients then received the opposite treatment for another 5 weeks. At this time, patients again reported to the blinded staff at University of Utah and repeated the exercise task.

### 2.4. Exercise Task

The acute exercise challenges were conducted during the fifth week of both treatment and placebo phases. The exercise task consisted of sustained (25 min) submaximal exercise using the Schwinn Air-Dyne bicycle ergometer. For the first exercise task in week 5, work-rate was gradually increased during the first 5 minutes until each subject attained a heart rate corresponding to 65–75% of age-predicted maximum heart rate. Thereafter, work-rate was adjusted as necessary to maintain the target heart rate. For the second exercise task (after 5 weeks on the second treatment, in week 12), we replicated the first exercise session so that work-rate was equivalent for both placebo and pregabalin conditions. Healthy controls completed one exercise session using the same protocol used for patients. During each exercise task, heart rate (HR) was recorded each minute, systolic blood pressure (SBP) and diastolic blood pressure (DBP) were recorded at minutes 10 and 20, and rating of perceived exertion (RPE) was recorded every 5 minutes using a modified Borg Scale [20]. Ratings of mental fatigue, physical fatigue, and pain using a 0–100 scale were provided by the patients and controls at baseline, in the middle of the exercise, after exercise, and at 0.5, 8, 24, and 48 hours after exercise.

### 2.5. Classification of Patients as Responders versus Nonresponders

Patients were classified as pregabalin responders or nonresponders prior to data analysis. Classification used the “Effects of Study Medication” questionnaire that patients completed after each treatment. The first question, “How satisfied are you with the study medication you have used most recently?,” was scored as 0 (not at all), 1 (somewhat), 2 (moderately), 3 (quite a bit), or 4 (very much). Then, patients circled symptoms that “got better” and symptoms that “got worse” from a list of 16 symptoms. We also recorded and tallied symptoms that were reported but not on the list.

### 2.6. Analysis

Initially, unpaired *t*-tests were utilized to determine whether FM and CFM + FM patients differed significantly for any exercise variable. Analyses of variance (ANOVAs) were used to compare controls to all patients while taking pregabalin and placebo. Repeated measures analyses with planned contrasts were used to examine treatment differences in exercise variables and subjective ratings of pain, fatigue, and exertion. Follow-up analyses examining responses in pregabalin responders and nonresponders were also performed. To ensure that differences in cardiovascular responses were not influenced by differences in physical work, these measures were adjusted for individual differences in work-rates. Significance was set at alpha <0.05.

## 3. Results

### 3.1. FM Compared to CFS + FM

The only variable that was significantly different between FM patients and CFS + FM patients was exercise work-rate (*p* = 0.03). FM patients achieved higher work-rates than CFS + FM patients on both placebo and pregabalin. On placebo, average work-rate for FM-only patients was 332 ± 58.7 kcal/hr while average work-rate was 252 ± 52.7 in CFS + FM patients. After pregabalin treatment, average work-rate was 328 ± 69.4 and 282 ± 56.6 kcal/hr for FM-only and CFS + FM patients, respectively. Data from these two groups were combined for subsequent analyses.

### 3.2. Descriptive Characteristics

A total of 20 patients (9 FM and 11 CFS + FM) volunteered to participate in the study. One FM patient refused to discontinue the original treatment and thus did not complete the second arm (placebo phase) of the protocol; this subject was excluded from analysis. Eighteen healthy controls served as a comparison group for the exercise task. As shown in Table 1, there were no significant differences for age or body mass index (BMI), between controls and all patients, or between pregabalin responders and nonresponders (*p* > 0.27). All groups were composed mostly of females.

Of the 19 patients completing both treatments, 6 scored 0 (not at all satisfied) on the first item of the “Effects of Study Medication” questionnaire and were classified as nonresponders. One patient who scored this item as 1 (somewhat satisfied) was also classified as a nonresponder based on the number of symptoms that got worse versus better on pregabalin (6 versus 3) while reporting 0 symptoms getting worse or better on placebo. Therefore, 12 were classified as pregabalin responders and 7 as nonresponders (see Table 2). Importantly, 6 of the FM patients and 6 of the CFS + FM patients were classified as pregabalin responders, while 2 FM and 5 CFS + FM patients were nonresponders. Thus, the FM and the CFS + FM patient groups had similar percentages of pregabalin responders (Fisher's Exact Test *p* = 0.633). Patients with orthostatic intolerance were also present in both responder and nonresponder groups (Table 1).

### 3.3. Effects of Pregabalin versus Placebo in Responders versus Nonresponders

Responders reported significantly higher treatment satisfaction and significantly more symptoms that improved during the pregabalin condition compared to placebo (*p* < 0.01, Table 2). All responders reported decreased pain, including muscle and joint pain, back pain, and headaches, and the majority also noted improved ability to do chores, total energy, and sleep, as well as decreased postexertional malaise. Additionally, responders reported significantly more symptoms that worsened while on placebo.

Nonresponders reported significantly lower treatment satisfaction during the pregabalin condition (*p* < 0.05) and mixed symptom responses during both conditions. Importantly, nonresponders reported more negative symptom responses while taking pregabalin compared to placebo, although this difference was only borderline significant (*p* = 0.06). Also, two nonresponders reported decreased muscle and joint pain while taking pregabalin, but their many negative symptoms outweighed the beneficial effects. The most common symptoms that became worse during pregabalin treatment were light-headedness (3 responders and 3 nonresponders) and mental fog (0 responders and 5 nonresponders).

### 3.4. Subjective Responses during and after Exercise

Average exercise work-rate did not differ between healthy controls and patients on either treatment (*p* > 0.11) (Table 3), indicating that the absolute intensity of the exercise task was equivalent among groups. This also suggests that underlying fitness levels were similar for patients and controls.

#### 3.4.1. Rating of Perceived Exertion (RPE)

**Figure 1:**
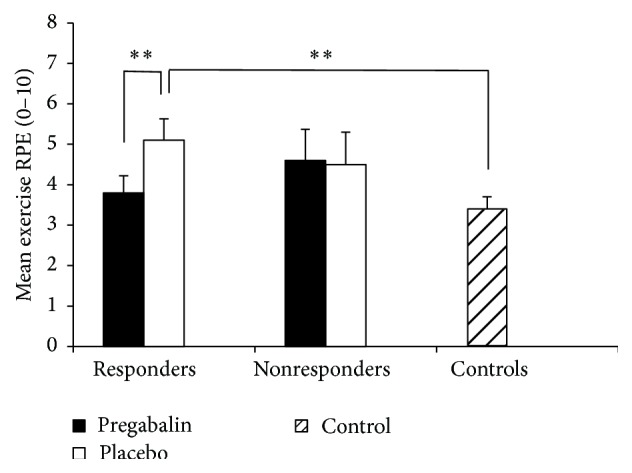
Rating of perceived exertion (RPE) in pregabalin responders, nonresponders, and controls. *∗∗* indicates significant difference, *p* < 0.01.

**Figure 2:**
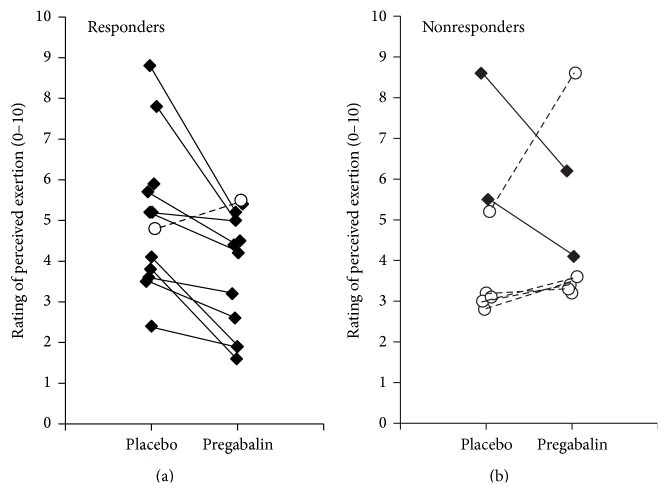
Individual ratings of perceived exertion (RPE) in pregabalin responders and nonresponders during pregabalin and placebo conditions.

When comparing all patients to controls, we observed that patients on placebo reported significantly higher average RPE during exercise compared to healthy controls (*p* = 0.011). In patients, average exercise RPE was significantly lower during the pregabalin condition compared to the placebo condition (*p* < 0.049, Table 3). Analysis of responders versus nonresponders showed that only responders had lower RPE during pregabalin treatment compared to placebo (*p* = 0.003). These ratings in responders on pregabalin were also similar to the RPE ratings of controls (*p* = 0.459, Figure 1). Conversely, for responders during placebo treatment, RPE was significantly higher than controls (*p* = 0.006). The nonresponders exhibited no treatment difference for RPE (pregabalin versus placebo, *p* = 0.842, Figure 1). All but one of the responders showed decreases in RPE during pregabalin treatment (Figure 2(a)), while only two of the seven nonresponders reported lower RPE while on pregabalin (Figure 2(b)).

#### 3.4.2. Ratings of Fatigue and Pain

**Figure 3:**
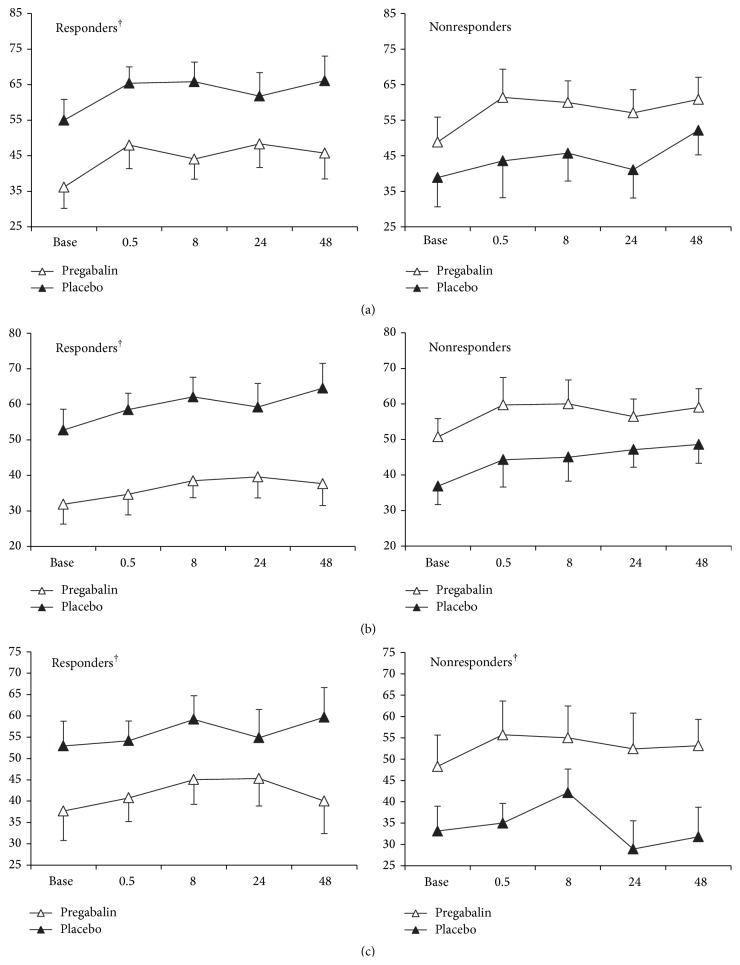
Subjective ratings of physical fatigue (a), pain (b), and mental fatigue (c) in responders and nonresponders during pregabalin and placebo treatments. Values are mean ± SE at baseline (preexercise) and at 0.5, 8, 24, and 48 hours after exercise. † indicates significant treatment effect across all time points, *p* < 0.05.

The patient group as a whole exhibited no treatment differences (pregabalin versus placebo) for physical fatigue, mental fatigue, or pain. When considered separately, responders had lower scores when on pregabalin versus placebo for mental fatigue (*p* = 0.030), physical fatigue (*p* = 0.009), and pain (*p* = 0.001, Figure 3). In nonresponders, mental fatigue was significantly higher during the pregabalin condition versus placebo (*p* = 0.030). Physical fatigue and pain in nonresponders also trended toward worsening with pregabalin versus placebo (*p* = 0.098 and 0.175, resp., Figure 3).

### 3.5. Cardiovascular Responses during Exercise

During pregabalin treatment, patients as a whole had significantly lower absolute HR and relative HR (% age-predicted maximal HR) during exercise compared to placebo (*p* = 0.024 and 0.029, resp.), despite the fact that exercise work-rate was slightly higher during the pregabalin condition (Table 3). Patients on pregabalin also had significantly lower absolute and relative exercise HR compared to controls (*p* < 0.002).

**Figure 4:**
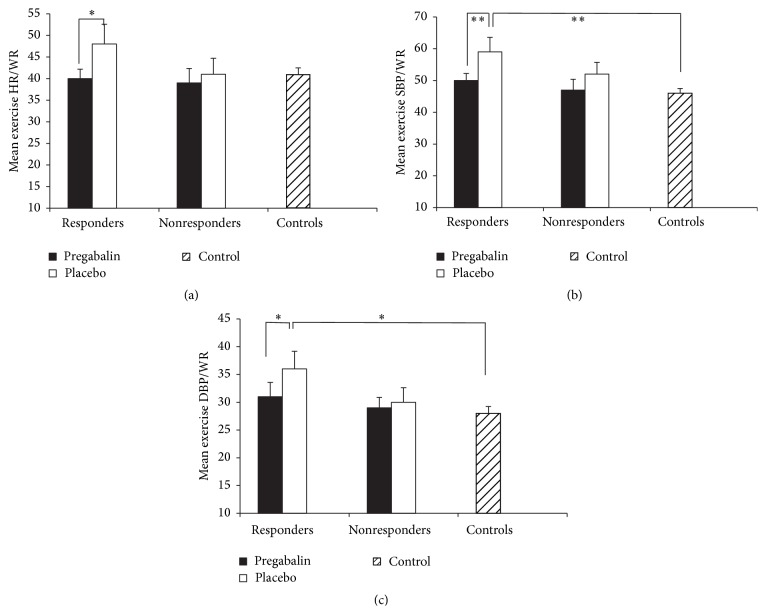
Exercise cardiovascular responses; heart rate (a), systolic blood pressure (b), and diastolic blood pressure (c) expressed relative to exercise work-rate in pregabalin responders, nonresponders, and controls. *∗* indicates significant difference, *p* < 0.05. *∗∗* indicates significant difference, *p* < 0.01.

When exercise HR was expressed relative to work-rate (HR/WR), HR/WR was significantly lower during pregabalin treatment compared to placebo in the whole patient group (*p* = 0.034). There were no differences in HR/WR between controls and patients on either treatment (*p* > 0.219, Table 3). In pregabalin responders, exercise HR/WR was significantly lower during pregabalin versus placebo treatment (*p* = 0.033, Figure 4). There was no significant treatment effect for HR/WR in nonresponders (*p* = 0.356).

Analysis of all patients revealed that unadjusted SBP was significantly lower during pregabalin treatment compared to placebo (*p* = 0.017, Table 3). In addition, SBP was significantly higher in all FM patients during the placebo condition compared to controls (*p* = 0.031). Although for unadjusted DBP there were no significant treatment effects or differences between patients and controls, DBP expressed relative to exercise work-rate (DBP/WR) was significantly lower during pregabalin treatment (*p* = 0.037), as was SBP/WR (*p* = 0.002). For the whole FM patient group, both SBP/WR and DBP/WR during the placebo condition were significantly higher than controls (*p* = 0.013 and 0.020, resp., Table 3).

Analyses of responders and nonresponders revealed that during the placebo condition SBP/WR and DBP/WR were higher than controls only in responders (*p* < 0.01, Figure 4). Pregabalin treatment significantly decreased SBP/WR and DBP/WR in responders (*p* = 0.002 and 0.027, resp., Figure 4) to levels that were similar to controls (*p* > 0.261). In nonresponders, there were no treatment effects (pregabalin versus placebo) for either SBP/WR or DBP/WR.

## 4. Discussion

To our knowledge, this is the first study demonstrating that pregabalin treatment (versus placebo) normalizes BP and HR responses to sustained moderate exercise in the subgroup of FM patients with a positive response to treatment. Also, pregabalin reduced these patients' exercise RPE and symptoms of pain and fatigue before and during exercise, as well as blunting their postexertional malaise for 48 hours after the task. In nonresponders, BP response was not affected, perceived exertion during exercise was not improved, and pre- and postexercise symptoms of pain and fatigue tended to worsen rather than improve with pregabalin. Strengths of this study include the randomized, double-blinded placebo-controlled crossover design and the statistical control for slight differences in exercise work-rate.

Classification of pregabalin responders was based on subjective rating of overall satisfaction and whether symptom improvements outweighed negative symptom responses. Consistent with prior research on pregabalin treatment in FM [21–24], decreased pain, including muscle and joint pain, back pain, and headaches, was the most commonly reported symptom improvement (reported by all 12 responders), with the second most common benefit being improved sleep (reported by 75% of the responders). In addition to these beneficial effects, pregabalin improved other symptoms that have not been previously studied. Specifically, several fatigue-related symptoms improved, with over 50% of responders reporting less postexertional malaise, more energy, and greater ability to do chores or have fun. Additionally, 25–49% of responders reported less physical fatigue and need for bed rest. Importantly, the presence of comorbid CFS in these FM patients was not associated with being a pregabalin nonresponder or with worsened side effects. It is possible that, in some FM patients, pregabalin has an independent beneficial effect on sensory pathways involved in fatigue pathways as well as pain.

The causes of chronic pain and fatigue in FM and CFS + FM are not well understood, but there may be common mechanisms underlying both symptoms, including increased signaling of pain and fatigue and sympathetic nervous system (SNS) dysregulation which may be a cause or effect of the increased signaling. FM has been described as a disorder characterized by enhanced SNS activity [2, 25, 26], but studies have reported both increased and blunted cardiovascular and catecholamine responses to exercise and other stressors [27, 28]. Our prior investigation noted that FM patients had lower baseline norepinephrine levels but greater blood pressure (BP) increases to postural and speech stressors than controls [29]. Blinded treatment with propranolol versus placebo normalized these SNS-related alterations and also significantly reduced pain ratings, supporting the interpretation that FM involves heightened SNS drive. Pregabalin decreases the release of several neurotransmitters including norepinephrine [30] and has been specifically linked to decreases in SNS activity and the inhibition of sympathetically maintained pain [31, 32]. This decrease in SNS activity may be an important factor in its clinical benefit for FM patients who are responders to pregabalin treatment. Our research group has also identified some of the molecular receptors responsible for neuronal signaling of pain and fatigue that appear to be dysregulated in CFS and FM, especially after a moderate exercise stress [17, 18]. These receptors, including alpha- and beta-adrenergic receptors, exhibit altered expression after exercise and thus may play a role in amplifying postexertional pain and fatigue.

Our findings showed that FM patients who were nonresponders to pregabalin differed physiologically as well as symptomatically from patients who were responders. Their HR and BP responses to exercise on either treatment regimen did not differ from controls and were not altered by pregabalin. Differences in cardiovascular responses to exercise from controls that were seen in the FM patient group as a whole were subsequently found to be due to those who were responders. Thus, there may be subgroups of FM patients who do versus do not exhibit the SNS dysregulation linked to altered cardiovascular exercise responses, and this dysregulation may be associated with response to pregabalin. Furthermore, on pregabalin, the symptom profile of nonresponders (including pre- and postexercise pain, physical fatigue, and mental fatigue ratings) actually tended to worsen, although the adverse effect was significant only for mental fatigue. This suggests that different physiological systems may be the primary ones that maintain the FM pain and other symptoms in this subgroup of patients. Further research is needed to identify what processes are dysregulated in these FM patients and to clarify treatments that may be more effective in reducing their pain without increasing side effects. Our findings further suggest that pretests of SNS function in untreated FM patients may be useful as biomarkers to help target the most effective pharmacotherapy for different subgroups of FM patients.

The decreases in exercise HR and BP responses during pregabalin treatment observed in responders may be a secondary effect of decreased pain, especially pain in muscles, joints, and connective tissue that are active during such exercise. Because fibromyalgia pain is associated with elevated inflammatory and stress markers [33, 34], this secondary effect might also act through decreased SNS activity or through altered inflammatory and immune effects, both of which could influence cardiovascular reactivity at rest and during exercise.

Our observations of blunted exercise HR and BP responses are consistent with pregabalin's use during presurgical intubation during which the hemodynamic pressor response is attenuated [35]. Alterations in feedback from group III and IV sensory afferents could contribute to this response. Normally, these afferents respond to metabolites produced during exercise and influence perceptions of fatigue and pain [36] and also increase sympathetic responses [37–39].

Although our findings clearly demonstrate that not all FM patients will benefit from pregabalin treatment, those who were responders reported improvement of pain symptoms, better sleep, and lower perceived exertion during exercise. These effects may have enhanced the ability to perform daily life activities as evidenced by these patients' reports of increased energy and ability to do chores or have fun and decreased physical and mental fatigue. If these symptom improvements result in less sedentary activity and participation in more active pursuits, health and quality of life could be improved. Although both aerobic and strength training regimens are among the best documented nonpharmacological interventions to reduce FM pain [10, 11], only a small percentage of FM patients are willing to undertake such training. Most patients are concerned that exercise will worsen their pain [15] which in turn may require increased bed rest and other limitations on function. Our findings suggest that FM patients who respond positively to pregabalin treatment may respond more positively to rehabilitative exercise. Potential benefits include reduced pain and perceived exertion during exercise and less postexertional worsening of pain and fatigue. Pregabalin pretreatment could thus remove a major barrier to physical training for many FM patients. This hypothesis could be directly tested by a 2-phase intervention study combining an initial phase of pregabalin versus placebo treatment with a second phase of exercise training.

*Limitations*. The primary weakness of this pilot study is the small sample size. This indicates the need for replication in a larger sample of people with FM and FM + CFS in order for the results to be generalizable to these populations.

## 5. Conclusion

FM patients who were responders to pregabalin were characterized by increased BP and HR responses to exercise compared to healthy controls. Pregabalin treatment normalized these cardiovascular responses to exercise and reduced perceived exertion in these patients. Nonresponders to pregabalin had normal exercise BP and HR responses on placebo, and these responses were not altered by pregabalin. In addition to expected benefits on pain and sleep quality, FM patients who were pregabalin responders demonstrated significant reductions in multiple fatigue-related symptoms, and the presence of comorbid CFS was* not* associated with nonresponse to pregabalin. Future research to determine whether pretreatment of FM patients with pregabalin could reduce initial postexertional symptoms and enhance success in completing an exercise training program is recommended.

## Figures and Tables

**Table 1:** Participant descriptive characteristics.

	Controls (*n* = 18)	All patients (*n* = 19)	Responders (*n* = 12)	Nonresponders (*n* = 7)
Age (yrs)	40.8 ± 2.8	43.4 ± 2.9	41.7 ± 3.2	46.3 ± 5.6
BMI (kg/m^2^)	27.7 ± 1.7	27.6 ± 1.4	28.9 ± 1.7	25.4 ± 2.4
Sex (M/F)	1/17	1/18	0/12	1/6
Dx (CFS + FM/FM)	na	11/8	6/6	5/2
Orthostasis	na	9	5	4

BMI: body mass index; Dx (CFS + FM/FM): proportion of patients with CFS + FM relative to FM only; Orthostasis: number of patients with orthostatic intolerance as determined by a score of 5 or greater on the Mayo Clinic Self-Report Orthostatic Grading Scale. Values are mean ± SE.

**(a):** 

	Responders (*n* = 12)			Nonresponders (*n* = 7)		
	Pregabalin	Placebo	Preg versus Plac	Pregabalin	Placebo	Preg versus Plac
Satisfaction	2.5 ± 0.37	0.3 ± 0.25	**<0.001**	0.1 ± 0.14	1.6 ± 0.57	**0.039**
# Sx improved	7.2 ± 1.35	1.3 ± 0.91	**<0.001**	1.0 ± 0.53	4.3 ± 2.11	0.106
# Sx worse	1.8 ± 0.52	6.4 ± 1.38	**0.002**	3.7 ± 0.99	1.9 ± 0.63	0.064

**(b):** 

	Responders (*n* = 12)		Nonresponders (*n* = 7)	
	Improved	Worse	Improved	Worse
Placebo condition				
≥50%	0	Sleep (7) Physical fatigue (7) Ability to do chores (7) Total energy (6) Muscle pain (6) Mental fog (6)	0	0
25–49%	0	Need for bed rest (5) Headaches (4) PEM (3) Sensitivity to noise (3) Ability to relax (3)	Mental fog (3) Pain (3) Ability to do chores (2) Sleep (2) Need for bed rest (2) Anxiety (2) Depression (2) Back pain (2) Ability to relax (2) Sensitivity to noise (2)	Sleep (2) Physical fatigue (2) Light-headedness (2)

Pregabalin condition				
≥50%	Muscle pain (10) PEM (8) Ability to do chores (8) Total energy (7) Sleep (7)	0	0	Mental Fog (5)
25–49%	Physical fatigue (5) Mental fog (4) Back pain (4) Headaches (4) Depression (4) Ability to relax (4) Need for bed rest (4)	Weight gain (3) Light-headedness (3)	Pain (2) Sleep (2) Total energy (2)	Light-headedness (3) Anxiety (2) Depression (2) Headaches (2) Blurred vision (2)

Satisfaction was rated on a 0-to-4 scale, where 0 means “not at all” and 4 means “very much.”

# Sx: number of symptoms reported by participants; values are mean ± SE.

≥50% refers to symptoms that were reported in at least 50% of the group.

25–49% refers to symptoms that were reported in 25–49% of the group.

PEM: postexertional malaise.

**Table 3:** Exercise task variables (mean ± SE) during pregabalin and placebo conditions in patients and healthy controls.

	Controls	All patients (*n* = 19)		All patients, *p* value		
	(*n* = 18)	Pregabalin	Placebo	Preg versus Con	Plac versus Con	Preg versus Plac
WR (kcal/hr)	318 ± 11.6	301 ± 14.9	286 ± 15.4	0.387	0.109	0.114
RPE	3.4 ± 0.3	4.1 ± 0.4	4.9 ± 0.4	0.172	**0.011**	**0.049**
HR (bpm)	127 ± 2.1	115 ± 2.5	122 ± 3.6	**0.001**	0.276	**0.024**
HR (% PMHR)	71 ± 0.7	65 ± 1.2	69 ± 1.8	**<0.001**	0.404	**0.029**
SBP (mm Hg)	142 ± 3.7	142 ± 3.8	153 ± 3.7	0.926	**0.031**	**0.017**
DBP (mm Hg)	86 ± 2.3	88 ± 2.6	91 ± 2.2	0.676	0.086	0.136
HR/WR	41 ± 1.5	40 ± 1.8	45 ± 3.2	0.606	0.219	**0.034**
SBP/WR	46 ± 2.3	49 ± 2.6	57 ± 3.4	0.345	**0.013**	**0.002**
DBP/WR	28 ± 1.2	30 ± 1.8	34 ± 2.3	0.229	**0.020**	**0.037**

WR: work-rate; RPE: rating of perceived exertion; HR: heart rate; PMHR: age-predicted maximal heart rate; SBP: systolic blood pressure; DBP: diastolic blood pressure; HR/WR, SBP/WR, and DBP/WR: values expressed relative to exercise work-rate (kcal/hr) multiplied by 100; Preg: pregabalin; Plac: placebo.
